# Trends and Pitfalls in the Progress of Network Pharmacology Research on Natural Products

**DOI:** 10.3390/ph18040538

**Published:** 2025-04-07

**Authors:** Alexander Panossian

**Affiliations:** Phytomed AB, Sjöstadsvägen 6A, 59344 Västervik, Sweden; ap@phytomed.se

## 1. Introduction

Herbs, used as food and a source of medicine for centuries, have been extensively studied over time for their chemical and pharmacological properties, with two main aims. One objective has been to identify and purify active compounds, which could then be developed into new, more active pharmaceuticals and analogs targeting specific receptors while being free of adverse effects. The other aim has been to combine several plant extracts or purified ingredients into new, multitarget hybrid combinations with enhanced pharmacological activity.

The first approach has been successfully applied in orthodox Western medicine, resulting in the discovery of compounds such as morphine, salicin, papaverine, and taxol, as well as their synthetic analogs. This approach has shaped the current drug development platform, which is primarily based on the pharmacodynamics and pharmacokinetics of botanicals. It also draws on the accrued knowledge of how biologically active molecules from plant extracts interact with receptor molecules, primarily proteins, involved in cellular and organismal responses to achieve therapeutic effects and maintain organismal homeostasis. Meanwhile, the stimulus–response coupling is often mediated by complex intra- and extracellular communications and feedback regulations within networks of signaling pathways and pharmacological systems, including the neuroendocrine–immune complex, cardiovascular, gastrointestinal, and other systems.

The one-drug-one-target paradigm, commonly explored for Galenic preparations by extracting one or more active constituents of a plant, was based on a reductionistic concept of dissecting biological systems into their constituent parts. However, even purified active compounds are not selective in their action, as they interact with other receptors and molecular networks associated with various functions, inducing adverse events.

In contrast, botanical hybrid preparations (BHP) [1] are designed to target multiple pathways, achieving therapeutic benefits in stress-induced, aging-related, and other chronic diseases [1,2,3,4,5]. When combining botanicals that contain numerous phytochemicals, the most desirable outcome can result in additional therapeutic benefits. However, the intended or expected outcomes can often be unpredictable, as various interactions occur within the target cells of the neuroendocrine–immune, cardiovascular, gastrointestinal, and other regulatory systems involved in the body’s response [1,2,3,4,5].

The second approach, primarily used in traditional Chinese medicine (TCM), is based on empirical knowledge and is undergoing modernization by incorporating current understanding of pharmacological systems and network pharmacology, including metabolomics, proteomics, transcriptomics, genomics, and microbiomes (collectively known as OMICS) [6,7,8,9,10].

Complex herbal combinations have a long history in traditional medical systems. Most traditional Chinese medicines contain more than one medicinal plant, suggesting that the concomitant use of additional plants may positively alter the effect of the primary herb [6]. Traditional Chinese medicine often provides multitarget (polyvalent) treatment, where ingredients address multiple symptoms and target various organs. In TCM, multi-component herbal preparations are formulated with the belief that each herb included in a prescription targets specific symptoms and that the overall mixture is more than a random assortment of herbs. Instead, the herbs interact harmoniously, each playing a distinct role in the hierarchy, where each herb is figuratively categorized as either “emperor”, “minister”, “assistant”, or “servant”. While the rationale of TCM may seem contrived when judged from a scientific perspective, its empirical effects are evident [6].

The resulting impact—whether complementary or antagonistic—is categorized into six basic modes of interaction: reinforcement (where herbs possessing similar medicinal properties are combined enhance efficacy), potentiation, restraint, detoxification, counteraction (where one herb reduces the therapeutic effect of another, representing an antagonistic interaction), and toxicity (though scientific evidence remains limited or lacking) [6].

## 2. Network Pharmacology of Natural Products and Complex Herbal Mixtures in Drug Discovery Programs

Historically, drug development strategies assumed that a single-target mechanism of action is the best approach for obtaining target-specific therapeutics. This model aims to treat specific conditions and is associated with fewer adverse events. However, many drugs and natural compounds interact with multiple receptors, resulting in polyvalent pharmacological and pleiotropic therapeutic activities through multitarget interactions. This development, in turn, has shifted the paradigm from a “one-target, one-drug” mode to a “network-target, multiple-component therapeutics” mode.

Developing techniques for monitoring the expression of genes, proteins, and metabolites in their entirety in cells, tissues, and organs formed the basis for the new field of systems biology. The aim of “*network pharmacology*” and “*network medicine*” is to understand the pathophysiology of diseases at the systems level. Disease-related interaction networks form the basis for developing novel drugs that target these networks, rather than individual proteins or enzymes [5].

The term “pharmacological target” refers to biomolecules such as DNA, mRNA, and proteins, including transmembrane and nuclear receptors, ion channels, transport proteins, and numerous enzymes to which a drug binds primarily to elicit its pharmacological effect. The complementary binding of drug molecules to a protein with a physiological function can alter the cell’s physiological responses, resulting in a pharmacological effect. The efficacy of binding a drug (ligand) depends on the drug’s chemical structure and affinity for its specific biomolecule (receptor).

The essence of *network pharmacology* is to evaluate how a study *drug* interacts with therapeutic targets, their associated signaling pathways involved in biological and physiological processes, and the functions linked to diseases, ultimately aiming to achieve a beneficial *therapeutic effect*.

The network pharmacology of a *multi-component drug substance*, which comprises several *phytochemicals* or *herbs/botanicals*, is more complex due to numerous challenges, including detailed chemical characterization (qualitative and quantitative analysis of phytochemical composition) and standardization, both of which are necessary to ensure *reproducible quality* and *efficacy*.

The pharmacology of natural products, particularly botanicals, encompasses pharmacognosy in Galenic preparations and the study of the pharmacodynamics and pharmacokinetics of their active constituents. Pharmacological studies aim to investigate the effect of herbal drugs and other botanicals on physiological systems to provide therapeutic benefits, focusing on the mechanisms related to pharmacological targets and the regulation of physiological processes.

Over the past two decades, we have witnessed a technical revolution, marked by novel methods collectively known as “omics” technologies. The term “omics”, derived from the Latin suffix “-ome”, refers to the measurement of large quantities to understand a biological question or phenomenon in an integrated manner [5,11,12]. Typical omics technologies include genomics, transcriptomics, proteomics, and metabolomics. More recently, additional “omics” methods have emerged, including epigenomics, lipidomics, and glycomics, as well as microbiomics in the gut, skin, and other organs, which are considered an “extended genome” because microorganisms contribute to the human genome [13].

As outlined above, traditional drug discovery programs focused on developing potent drugs with some specificity for a particular therapeutic target. However, due to the promiscuity of many medicines, which can modulate multiple targets and pathways [14,15], the term “polypharmacology” has been introduced. Bioinformatic tools now have the power to design multi-specific drugs [16,17,18,19]. Thus, drug promiscuity has shifted from being seen as the source of unwanted effects to becoming an intentional strategy in drug discovery and development. Network pharmacology can now integrate complex “omics”-based datasets from “multi-omics” and single-cell “omics” technologies to form the foundation for novel next-generation drugs. Notably, medicinal plants and their associated phytochemicals play a pivotal role in this emerging field of drug research.

Network pharmacology has been applied to various traditional medicines, with the most progress being made in the context of traditional Chinese medicine (TCM) [7,8,20]. Network pharmacology aligns perfectly with the holistic philosophy of TCM. While a body of clinical data reports the efficacy of TCM, the mechanistic understanding of herbal TCM formulations is still in its infancy [20,21,22,23,24]. The expectation is that the theory of TCM, which fundamentally differs from the reductionist approach of Western medicine, could eventually be scientifically explained [9,10,25]. Network-based multitarget-multicomponent models could help rationalize the understanding of characteristic TCM syndromes [26]. In TCM, a syndrome describes an imbalance in the body that can lead to various diseases; for example, hot or cold syndromes result in diseases related to excess heat or cold in the body [27].

Network pharmacology, a relatively new discipline, has emerged recently and is rapidly developing [5,28,29,30,31]. In 2024 alone, approximately 9000 publications on network pharmacology from around the world were cited and indexed in the PubMed database [32]. In particular, the advancement of integrative omics network pharmacology and artificial intelligence in natural products has opened up new avenues for the following areas:

- elucidation of the mechanisms of action of medicinal plants [3,33,34,35,36,37,38,39];
- understanding the synergistic therapeutic actions of complex bioactive components in medicinal plants [1,2,3,4,5,40,41];
- providing a rationale for traditional Chinese medicine, as well as enhancing the quality of TCM drug research and the speed and efficiency of developing new TCM products [7,8,9,10,42,43];
- discovering and developing new botanical hybrid combinations [1,2,5];
- predicting drug–herb interactions [44], adverse events [45] and potential toxic effects [12,46,47,48].

## 3. Critical Appraisal, Limitations, Challenges, and Future Perspectives

Since botanicals consist of multi-component active compounds and phytochemicals, their interactions lead to novel and unexpected pharmacological activities due to their synergistic and antagonistic effects. Similarly, the pharmacological activity of a combination of several plants or herbal extracts—such as those in botanical hybrid preparations (e.g., TCM formulas)—results in a new biologically active substance with unique pharmacological characteristics [1]. However, there are several challenges in network pharmacology for natural products research:

(I) The reproducibility of the chemical composition (fingerprint) of active compounds and their influence on the pharmacological activity (signature) of the total extract is crucial. This is due to the synergistic, potentiating, and antagonistic interactions between the multiple targets of the numerous active components in the extracts.
(II) Quality and safety issues related to the content of active and potentially toxic compounds should be considered [49]. European drug legislation and pharmacopeia specify active markers and their content in herbal medicines and botanical preparations, ensuring reproducible quality, safety, and efficacy. However, most publications on TCM do not comply with EC standards, particularly when assessing TCM in clinical trials. This is a significant obstacle in determining endpoint outcomes and drawing conclusions regarding the reproducible quality, safety, and efficacy of TCM.
(III) The optimal effective and safe therapeutic dose of *herbal medicines and botanical preparations* should be established, taking into account the “bell-shaped” dose–response relationship. Many in vitro studies have applied supraphysiological concentrations far exceeding the proposed doses in humans. Most studies have overlooked this issue, as pharmacological and toxicological data are often unavailable. The optimal range of doses for botanicals is similar to that of the hormetic zone dose-response pattern. The underlying molecular mechanisms of the reversal of the dose response are not fully understood. The theoretical background of hormesis is related to the hypothesis of interactions between biologically active compounds (ligands, interventions, or drugs) and two target proteins (receptors) that exhibit functionally opposite responses at different concentrations. It has been proposed that a drug acting as a competitive antagonist at either or both receptors changes the relationship between the two opposing concentration–effect curves, resulting in potentiation, antagonism, or reversal of the observed effect. The theoretical model suggests that the total effect in the system can be obtained by the algebraic summation of the two effects produced by the activation of the two opposing receptor populations, generating a classical biphasic hormesis curve. However, in practice, dose-response patterns are significantly complicated due to many other interactions with (i) multiple targets (receptors) with different affinities for the active compound, (ii) other regulatory proteins or mediators within the networks involved in the adaptive stress response, (iii) feedback downregulation within molecular signaling pathways, and/or (iv) the metabolic transformation of active ligands into metabolites, which may act as secondary ligands with varying affinities for different receptors in the adaptive stress response.
(IV) The network analysis model often limits itself to intracellular communications and overlooks extracellular communications and interactions between various physiological regulatory systems, such as the integrative nervous, endocrine, immune, cardiovascular, respiratory, and renal systems. These systems operate across multiple organs and tissues. In this mechanistic model, there is little room for considering the role of physiology and anatomy in the organism’s integrative regulatory system, which maintains homeostasis in response to pharmaceutical interventions. The primary challenge in network pharmacology is the lack of sufficient, evidence-based knowledge regarding the molecular biology, pharmacodynamics, and pharmacokinetics of all compounds in botanical preparations, as well as the interactions of mediators that influence their pharmacological activity. While bioinformatics involves building and mechanistically analyzing networks using available in silico tools, artificial intelligence alone can sometimes be insufficient. Valid experimental data, coupled with well-designed preclinical and clinical studies of well-characterized botanical preparations, are needed to ensure the established quality, efficacy, and safety of these compounds.
(V) The conclusions of many network analysis studies go too far and are not supported by evidence in many aspects. In such cases, further studies are often based on misleading backgrounds and can yield inconclusive results. Furthermore, referring only to the latest publications—mainly review articles—and ignoring original studies distorts the scientific history of many inventions.
(VI) Network pharmacology predictions based exclusively on in silico and some in vitro studies have several limitations that must be further validated through animal experiments.
(VII) Scientific information on the direction of correlations between gene expression and physiological function or diseases is used in silico for therapeutic efficacy and toxicity predictions. Additional studies are required where different experimental outcome measures are applied. Furthermore, a strong resistance mechanism can override the effects of a drug. For instance, the drug efflux transporter P-glycoprotein can expel medications from cells before they reach their intended intracellular targets, thereby preventing or reducing most downstream signaling and network effects. Ultimately, clinical studies on predicted diseases in human subjects are essential.
(VIII) One challenge lies in the compatibility of pharmaceutical-grade herbal extracts of reproducible quality and pharmacological activity compared to purified active constituents. The content of a genuine extract is adjusted to a defined content or range of active constituents with a known therapeutic efficacy or content of analytical markers.
(IX) Implementing Lipinsky’s so-called “rule of five” is not a relevant tool for screening traditional herbal medicines for new drug discovery.

In conclusion, the network pharmacology approach is highly useful for understanding the mechanisms of action, predicting potential toxic effects, and identifying new therapeutic indications. However, the results of in silico analyses must be validated with experimental findings, including gene and protein expression in isolated cells, and at least confirmed through in vivo animal experiments [49]. A deeper understanding of the molecular mechanism and well-designed preclinical and clinical studies of BHPs would pave the way for advancing the wealth of empirical knowledge in phytomedicine research.

The protein kinase B (AKT) is central in modulating GPCR-mediated PI3K/AKT canonical signaling pathways, which play a role in adaptive stress responses associated with many aging-related diseases and disorders. These pathways also regulate the neuroendocrine-immune and cardiovascular-respiratory systems [2,3,4,5,50]. Activating the intracellular PI3K/AKT-mediated signaling pathway triggers a cascade of reactions downstream of proteins, which mediate various cellular functions, including neurogenesis, angiogenesis, neuroinflammatory responses, and other repair mechanisms in many diseases [2,5,30,51,52,53,54,55]. A plethora of plant secondary metabolites target various biased GPCRs, signaling non-specifically to modulate the PI3K/AKT pathway in multiple organs and tissues, thereby regulating compromised cellular and systemic homeostasis and contributing to the resolution of acute and chronic neuroinflammatory disorders and diseases [2,5,30,51,52,53,54,55]. Different types of phytochemicals collectively contribute to various therapeutic actions that target this mediatory intracellular system [2,5,30,51,52,53,54,55]. In this context, further network pharmacology studies of botanicals and BHP are required, with a focus on establishing relationships between distinct phytochemical types of compounds and specific indications for various diseases.

In this Special Issue, we recommend adhering to the commonly accepted terms and definitions below to avoid misuse of terminology (Table 1).

## Figures and Tables

**Table 1:** Glossary, terminology, and definitions.

Terms	Definitions
*Biased ligand*	Biased ligands are those that can regulate receptor activity by stabilizing distinct conformations. Since diverse signaling pathways elicit distinct physiological effects, biased ligands that selectively induce beneficial pathways hold promising therapeutic value. Ligand bias refers to the ability of ligands to activate specific receptor signaling responses more effectively than others. It reflects differences in the reactions of a receptor to specific ligands and has implications for the development of highly specific therapeutics.
*Biased signaling*	Biased signaling is a natural consequence of GPCR or allosteric function and should be expected from both natural agonists and synthetic analogs. Biased signaling of G protein-coupled receptors or receptor tyrosine kinases describes the ability of different ligands to activate an alternative downstream signaling pathway preferentially.
*Botanicals*	Products that include plant materials, algae, macroscopic fungi, and combinations thereof.
*Botanical hybrid preparations/products*	The term “botanical hybrid preparation” (BHP) describes the pharmacological activity of a fixed herbal combination with a specific chemical composition.
*Herbal substance*	All herbal substances are mainly whole, fragmented, or cut plants, plant parts, algae, fungi, and lichen, typically in an unprocessed, often dried form, though some many be used fresh. Herbal substances are precisely defined by the plant part used and the botanical name, following the binomial system (genus, species, variety, and author).
*Herbal preparations*	Herbal preparations are obtained by subjecting herbal substances to various treatments, including extraction, distillation, expression, fractionation, purification, concentration, and fermentation. These include comminuted or powdered herbal substances, tinctures, extracts, essential oils, expressed juices, and processed exudates.
*Herbal medicinal products*	Any medicinal product exclusively containing as active substances one or more herbal substances, one or more herbal preparations, or one or more such herbal substances combined with one or more such herbal preparations.
*Pharmacognosy*	A multidisciplinary subject that encompasses aspects of botany, organic chemistry, biochemistry, and pharmacology, focusing on natural products used in the production and discovery of new drugs.
*Pharmacophore*	The distinct functional groups or substance classes that possess the biological activity necessary to ensure optimal interaction between their receptor and a specific biological target structure and to trigger (or block) their biological response.
*Pharmacodynamics*	Pharmacodynamics refers to the effects of drugs on the body, including receptor binding, post-receptor effects, and chemical interactions.
*Pharmacokinetics*	Pharmacokinetics describes how the body affects a drug’s absorption, distribution, metabolism, and excretion after administration.
*Pleiotropic action*	In pharmacognosy and ethnopharmacology, pleiotropic action refers to all actions of botanical preparation other than those for which it was explicitly intended. It is often used to denote additional beneficial effects but can also indicate detrimental adverse effects.
*Polypharmacology*	Polypharmacology refers to the binding of a drug to multiple target proteins, with clinical effects being mediated through the modulation of the set of protein targets.
*Polyvalent action:*	Polyvalence refers to the range of biological activities a botanical preparation may exhibit that contribute to the overall effect observed clinically or in vivo. Polyvalence can result from the following factors: o Various phytochemicals are present, with each type having a different biological effect; o Compounds of a single phytochemical type often exhibit multiple biological effects relevant to treating the disease and improving the patient’s health; o Compounds are present that do not affect the cause or symptoms of the disease but modify the absorption, distribution, metabolism, and excretion of active constituents, as well as the adverse effects.
*Rule of five*	Poor absorption or permeation of a compound is more likely when there are >5 hydrogen bond donors, the molecular mass is >500, cLogP is >5, and the sum of nitrogen and oxygen atoms in a molecule is greater than 10. Many drugs, however, are exceptions to the rule of five, and these are often substrates for biological transporters.
*Selective action*	o A drug strongly prefers its intended target over other targets; o The degree to which a drug acts on a given site relative to others; o The ability of a drug to affect a particular gene, protein, or signaling pathway in preference to others; o A drug that binds to the same receptor as an agonist but produces an effect opposite to that of the agonist.
*Specific action*	An accurate, exact (free from ambiguity), and distinctive influence specifically adapted to a purpose or use.
*Systems pharmacology*	It aims to comprehend how drugs interact with the human body as a complex, integrated biological system. Instead of considering the effect of a drug to be the result of a single specific drug–protein interaction, systems pharmacology refers to the impact of a drug as the outcome of the network of interactions it may have. Interaction networks may include chemical–protein, protein–protein, genetic, signaling, and physiological (at the cellular, tissue, organ, and whole-body levels). Systems pharmacology uses bioinformatics and statistical techniques to integrate and interpret these networks.
*Network pharmacology*	Network pharmacology is a subfield that combines principles from pharmacology, systems biology, and network analysis to study the complex interactions between drugs and their targets, such as receptors or enzymes, within biological systems. The topology of a biochemical reaction network determines the shape of the drug dose–response curve and the type of drug–drug interactions, thus helping design efficient and safe therapeutic strategies. The topology of network pharmacology utilizes computational tools and network analysis algorithms to identify drug targets, predict drug–drug interactions, elucidate signaling pathways, and explore the polypharmacology of drugs.
*Synergy*	Two or more agents work together to produce a result that cannot be achieved by any of the agents independently, resulting in the generation of new pharmacological activity specific to the combination of two or more agents. Another definition of synergy is broadly interpreted as the combination of agents that is more effective than the sum of their individual effects, without specifying the differences between potentiation and amplification.
*Systems biology*	Systems biology involves computational and mathematical analysis and modeling of complex biological systems. It is a biology-based interdisciplinary field that focuses on the complex interactions within biological systems and uses a holistic approach to biological research.
